# Cholecalciferol supplementation and angiogenic markers in chronic kidney disease

**DOI:** 10.1371/journal.pone.0268946

**Published:** 2022-06-03

**Authors:** Jaskiran Kaur, Kajal Kamboj, Ashok Kumar Yadav, Prabhjot Kaur, Vivek Kumar, Vivekanand Jha

**Affiliations:** 1 Department of Experimental Medicine and Biotechnology, Postgraduate Institute of Medical Education and Research, Chandigarh, India; 2 Department of Nephrology, Postgraduate Institute of Medical Education and Research, Chandigarh, India; 3 George Institute for Global Health, UNSW, New Delhi, India; 4 School of Public Health, Imperial College, London, United Kingdom; 5 Manipal Academy of Higher Education, Manipal, India; PLOS, UNITED KINGDOM

## Abstract

Vitamin D plays an important role in proliferation and differentiation of cells and deficiency of vitamin D disturbs angiogenic balance. Previous studies in animal models have reported an association between serum levels of vitamin D and balance between pro- and anti-angiogenic factors. There is insufficient evidence about the effect of vitamin D on mediators of angiogenesis in patients with CKD. We investigated the effect of cholecalciferol supplementation on serum levels of angiogenic markers in non-diabetic patients with CKD stage 3–4. In this secondary analysis on stored samples of our previously published randomized, double-blind, placebo-controlled trial, stable patients of either sex, aged 18–70 years, with non-diabetic CKD stage 3–4 and vitamin D deficiency (serum 25-hydroxyvitamin D ≤20 ng/ml) were randomized to receive either two directly observed oral doses of cholecalciferol (300,000 IU) or matching placebo at baseline and 8 weeks. The primary outcome was change in brachial artery flow-mediated dilatation at 16 weeks. Changes in levels of serum angiogenesis markers (angiopoietin-1, angiopoietin-2, VEGF-A, VEGEF-R, and Tie-2) between groups over 16 weeks were compared. A total 120 patients were enrolled. Supplementation with cholecalciferol led to significant improvement in FMD. Serum 25(OH)D levels were similar in both groups at baseline (13.21±4.78 ng/ml and 13.40±4.42 ng/ml; p = 0.888). At 16 weeks, the serum 25(OH)D levels increased in the cholecalciferol group but not in the placebo group (between-group difference in mean change:23.40 ng/ml; 95% CI, 19.76 to 27.06; p<0.001). Serum levels of angiogenic markers were similar at baseline. At 16 weeks, angiopoietin-2 level decreased in cholecalciferol group (mean difference:-0.73 ng/ml, 95%CI, -1.25 to -0.20, p = 0.002) but not in placebo group (mean difference -0.46 ng/ml, 95%CI, -1.09 to 0.17, p = 0.154), however there was no between-group difference at 16 weeks (between-group difference in mean change: -0.27 ng/ml, 95%CI, -1.09 to 0.55, p = 0.624). Serum angiopoietin-1 level increased [mean change: 5.63 (0.51 to 10.75), p = 0.018] and VEGF-R level decreased [mean change: -87.16 (-131.89 to -42.44), p<0.001] in placebo group but did not show any change in cholecalciferol group. Our data shows the changes in Ang-1, Ang-2 and Ang-1/Ang-2 ratio after high dose oral cholecalciferol supplementation in patients with non-diabetic G3-4 CKD. The data suggests changes in circulating levels of angiogenic markers which needs to be confirmed through an adequately powered study.

## Introduction

Angiogenesis, a physiological process that generally refers to the development of new blood vessels from pre-existing vessels [1], involves migration and proliferation of endothelial cells, lumen formation, maturation, and remodelling and is affected by a number of factors [2]. The balance between pro-angiogenic and anti-angiogenic molecules control the process of angiogenesis and imbalance of angiogenesis-related factors is implicated in the pathogenesis of various disorders like cancer, cardiovascular disease, kidney disease, and autoimmune diseases [3]. Many strands of evidence indicate that along with angiogenesis factors like angiopoietin-1(Ang-1), angiopoietin-2 (Ang-2), vascular endothelial growth factor-A (VEGF-A), vascular endothelial growth factor receptor (VEGFR), and Tie-2, vitamin D also plays a role in the regulation of angiogenesis [4]. Experimental studies have demonstrated an association between the balance of angiogenesis-related factors and the progression of CKD [5, 6].

Vitamin D plays an important role in the proliferation and differentiation of cells by affecting the balance of angiogenesis-related factors. Previous studies have demonstrated that 1,25(OH)_2_D_3_ has antiangiogenic properties *in vivo* and *in vitro*, and inhibits angiogenesis in a tumor model [7]. Vitamin D supplementation studies showed protective effects on the incidence of preeclampsia by promoting angiogenesis in endothelial progenitor cells [8, 9]. Vitamin D deficiency may disturb the angiogenic balance by increasing the production of angiotensin II, which further stimulates expression of Ang-2 and inhibits Ang-1 and Tie-2 leading to endothelial rarefaction in vitamin D deficient CKD [10, 11]. Futrakul et.al. reported a reduction in levels of serum VEGF-A and Ang-1, and high Ang-2 levels in patients with CKD [12]. Another observational study demonstrated that the imbalance between Ang-1 and Ang-2 (high Ang-2 and low Ang-1) was associated with a subclinical cardiovascular abnormality in patients with CKD [13].

Not much is known about the effect of vitamin D on mediators of angiogenesis in vitamin D-deficient patients with CKD. In a prespecified secondary analysis of randomized, double-blind, placebo-controlled trial of cholecalciferol supplementation in vitamin D-deficient, nondiabetic subjects with stage 3–4 CKD, we investigated the effect of oral cholecalciferol supplementation on serum levels of various angiogenic markers.

## Materials and methods

The current study is a secondary analysis on stored biological samples of a parallel-arm, randomized, double-blind, placebo-controlled trial that investigated the effect of a high dose of oral cholecalciferol supplementation on vascular function [14]. The study was done at the Postgraduate Institute of Medical Education and Research (PGIMER), Chandigarh, India. Ethics approval was obtained from Institute Ethics Committee to use the stored biological samples of the trial for biomarker analysis. Prior provided written informed consent was taken from all study subjects. The authors confirm that all ongoing and related trials for this drug/intervention are registered. The detailed study protocol and subject enrolment process including the Consolidated Standards of Reporting Trials (CONSORT) flow diagram have been described earlier [14]. Briefly, 120 non-diabetic, CKD stage 3–4 subjects, age between 18 to 70 years and serum 25(OH)D levels ≤20 ng/ml were included in this study. A computer generated Bernoulli random number table was used for randomization of study subjects. Randomization was done in a 1:1 allocation ratio to receive two directly observed doses of either 300,000 IU of cholecalciferol or matching placebo at baseline and 8 weeks later. Follow up was done at 16 weeks after randomization. The time gap between cholecalciferol doses and analysis of variables is concordant with what has already been published [15].

Demographic, clinical, and biochemical parameters were recorded at baseline and 16 weeks. Flow-mediated dilatation was assessed at baseline and 16 weeks using a Philips IU22 xMatrix ultrasound system (Philips, Cambridge, MA) and automated continuous edge detection and wall tracking software (Brachial Analyzer for Research 6, Medical Imaging Applications LLC, Coralville, IA). Angiogenic biomarkers were analysed at baseline and 16 weeks in serum samples. Serum 25(OH)D and 1,25(OH)_2_ D was measured by enzyme immunoassay [EIA; Immunodiagnostic Systems (IDS), UK], as described earlier [14]. Serum levels of Ang-1, Ang-2, vascular endothelial growth factor (VEGF), vascular endothelial growth factor receptor (VEGFR), and Tie-2 receptor were analysed using Quantikine ® ELISA kit (R&D Systems, USA) as per manufactures protocol.

### Outcomes

We compared changes in levels of angiogenesis biomarkers—Ang-1, Ang-2, VEGF, VEGFR, and Tie-2 receptor at 16 weeks from baseline after cholecalciferol supplementation.

### Statistical analysis

Data in this study are presented as mean with standard deviation (SD), mean change (95% confidence interval) and frequency (percentage) as appropriate. Normally distributed continuous variables were compared with independent samples Student’s *t* test, or Mann-Whitney *U* test. Categorical variables were analysed by Chi-Square or Fisher’s Exact tests as appropriate. Paired Student’s *t*-test and Wilcoxon signed-rank test were used for within-group comparisons for normally and non-normally distributed data, respectively. P-values <0.05 with two tailed were considered significant. Statistical Package for the Social Sciences (SPSS) software for Macintosh, version 21.0 (IBM Corp., Armonk, NY, USA) was used for all the statistical analysis.

## Results

**Fig 1:**
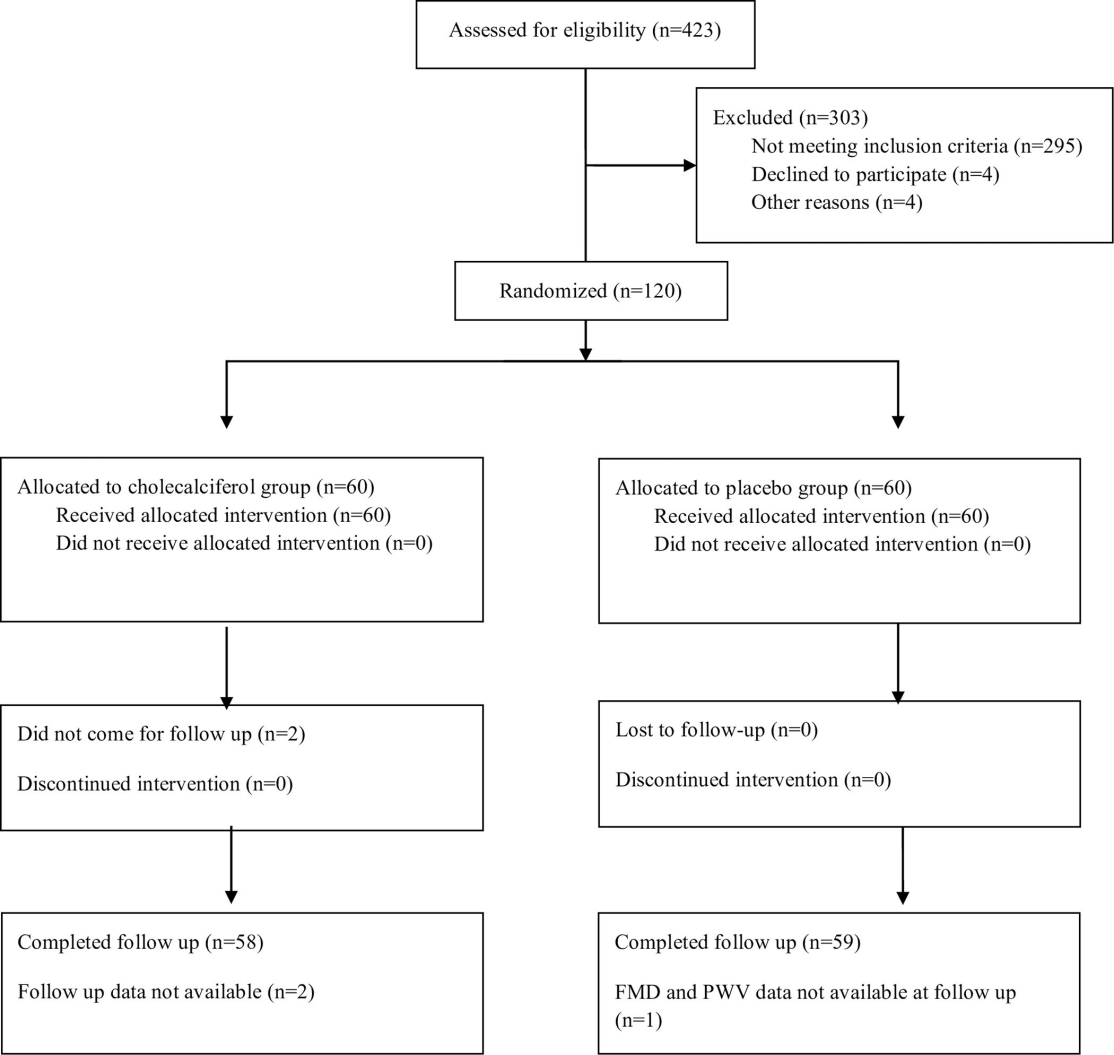
CONSORT flowchart.

A total of 423 subjects were screened in this study and 120 were enrolled, randomized after exclusion of 303 subject due to various reasons (Fig 1). Out of 120 randomized patients, 59 and 58 patients in the placebo and cholecalciferol group respectively completed their follow up. The baseline demographic characteristics of the two groups are shown in Table 1. Participant demographics and causes of CKD were similar in both groups. Biochemical parameters and serum level of angiogenic markers were also similar in both groups at baseline (Table 2).

**Table 1:** Baseline characteristics of study subjects.

Parameters	Placebo (n = 59)	Cholecalciferol (n = 58)	P value
Gender (M/F)	40/19	41/17	0.845
Age (years)	45.20±11.61	43.17±11.79	0.400
Body mass index (kg/m^**2**^)	23.45±2.91	23.57±2.67	0.818
SBP (mmHg)	127.88±15.67	128.48±14.43	0.971
DBP (mmHg)	82.33±10.21	83.38±10.09	0.902
Fasting blood sugar (mg/dl)	93.17±11.73	92.00±13.11	0.660
Duration of disease (months)	32.30±46.94	39.53±49.71	0.415
Number of subjects with proteinuria	30 (51)	31(53)	0.778
Hypertension	54 (92)	53 (91)	0.542
Family history of CAD	5 (8)	6 (10)	0.751
Family history of diabetes	17 (29)	14 (24)	0.531
Family history of kidney disease	7 (12)	8 (14)	0.782
**Cause of CKD**			
Chronic interstitial nephritis	11(19)	10 (17)	0.810
Chronic glomerulonephritis	5 (8)	6 (10)	1.000
Polycystic kidney disease	6 (10)	6 (10)	1.000
Unknown	30 (51)	27 (47)	0.583
Others	7 (12)	9 (16)	0.306

Data presented as mean± standard deviation and number (percentage).

SBP; systolic blood pressure, DBP; diastolic blood pressure, CAD; coronary artery disease, CKD; chronic kidney disease.

**Table 2:** Baseline level biochemical and circulating biomarkers in study subjects.

Parameter	Placebo (n = 59)	Cholecalciferol (n = 58)	P values
Haemoglobin (g/dl)	12.02±1.94	11.97±1.69	0.878
eGFR (min/ml/1.73m^**2**^)	34.63±12.25	35.77±12.37	0.723
Albumin (g/dl)	4.62±0.63	4.74±0.54	0.261
Calcium (mg/dl)	9.09±0.94	9.01±0.73	0.630
Inorganic phosphorus (mg/dl)	3.60 (3.07, 4.40)	3.58 (3.01, 4.08)	0.316
Uric acid (mg/dl)	7.66±2.37	8.01±2.32	0.435
25(OH)D (ng/ml)	13.86 (9.19, 17.79)	14.48 (9.34, 17.08)	0.888
1,25 (OH)_**2**_ D(pg/ml)	17.32 (10.58, 27.02)	18.13 (11.58, 23.69)	0.976
Ang-1 (ng/ml)	2.82 (0.90, 10.86)	5.73 (1.62, 10.44)	0.558
Ang-2 (ng/ml)	1.39 (0.92, 2.69)	1.52 (0.90, 2.39)	0.777
VEGF (pg/ml)	57.20 (17.40, 114.36)	61.10 (26.57, 87.42)	0.935
VEGFR (pg/ml)	400 (331, 494)	387 (335, 515)	0.931
Tie-2 (ng/ml)	20.71 (16.85, 24.87)	20.86 (15.08, 27.08)	0.881
Ang-1/Ang-2 ratio	1.50 (0.83, 5.80)	3.20 (1.01, 8.68)	0.185

Data presented as mean± standard deviation and median (25^th^, 75^th^ percentile).

eGFR; estimated glomerular filtration rate, 25(OH)D; 25 hydroxy vitamin D, 1,25 (OH)_2_ D; 1,25 di-hydroxy vitamin D, Ang-1; Angiopoitein-1, Ang-2; Angiopoitein-2 VEGF; vascular endothelial growth factor-A, VEGFR; vascular endothelial growth factor receptor, Tie 2; tyrosine kinase receptor-2.

### Change in biochemical parameters, endothelial function, and angiogenic biomarkers

Levels of serum 25(OH)D were similar in both groups at baseline (13.21±4.78 ng/ml and 13.40±4.42 ng/ml; p = 0.88). Serum 25(OH)D levels were increased in cholecalciferol group (mean change: +24.91 ng/mL; 95% CI: 21.77 to 28.06 ng/mL; p < 0.001) but not in the placebo group (mean change: +1.51 ng/mL; 95% CI: –0.46 to 3.48 ng/mL; p  =  0.130) at 16 weeks. There was significant between-group difference in mean change (23.40, 95% CI; 19.76 to 27.06, p<0.001; Table 3) in 25(OH)D levels at 16 weeks. Similarly, there was a rise in the serum 1,25 (OH)_2_ D levels in the cholecalciferol group, whereas the placebo group did not show any change. The difference in mean change in the levels of 1,25 (OH)_2_ D between the two groups was significant (between-group difference in mean change: 14.98 ng/ml; 95% CI: 4.48 to 27.18; p = 0.001; Table 3). At 16 weeks, the FMD improved in the cholecalciferol group but not in the placebo group [14].

**Table 3:** Change in serum levels of vitamin D and angiogenic marker at 16 weeks follow-up.

	Placebo (n = 59)		Cholecalciferol (n = 58)		Between group difference	
	Mean change (95% CI)	*p* value	Mean change (95% CI)	*p* value	Difference of Mean change (95% CI)	*p* value
25(OH)D (ng/ml)	1.51 (-0.46 to 3.48)	0.130	24.91 (21.77to 28.06)	**<0.001**	23.40 (19.76 to 27.06)	**<0.001**
1,25 (OH)_**2**_ D (pg/ml)	0.48 (-4.65 to 5.62)	0.608	15.46 (5.42 to 25.50)	**<0.001**	14.98 (4.48 to 27.18)	**0.001**
Ang-1 (ng/ml)	5.63 (0.51 to 10.75)	**0.020**	1.36 (-2.39 to 5.11)	0.280	-4.27 (-10.56 to 2.01)	0.445
Ang-2 (ng/ml)	-0.46 (-1.10 to 0.18)	0.154	-0.73 (-1.25 to -0.20)	**0.002**	-0.27 (-1.09 to 0.55)	0.624
VEGF (pg/ml)	34.11 (-66.11 to 134.35)	0.592	20.77 (-046.68to 88.24)	0.335	-13.34 (-133.38 to 106.60)	0.990
VEGFR (pg/ml)	-87.16 (-131.89 to -42.44)	**<0.001**	-69.77 (-142.74 to 3.20)	0.061	17.39 (-67.47 to 102.25)	0.985
Tie-2 (ng/ml)	0.14 (-3.28 to 3.55)	0.686	3.70 (-1.95 to 9.36)	0.427	3.56 (-2.98 to 10.12)	0.366
Ang-1/Ang-2 ratio	5.5 (1.73 to 9.31)	**0.003**	2.19 (2.30 to 6.68)	0.049	-3.32 (-9.12 to 2.48)	0.563

Data presented as mean change (95% CI).

25(OH)D; 25 hydroxy vitamin D, 1,25 (OH)_2_ D; 1,25 di-hydroxy vitamin D, Ang-1; Angiopoitein-1, Ang-2; Angiopoitein-2 VEGF; vascular endothelial growth factor-A, VEGFR; vascular endothelial growth factor receptor, Tie 2; tyrosine kinase receptor-2.

**Fig 2:**
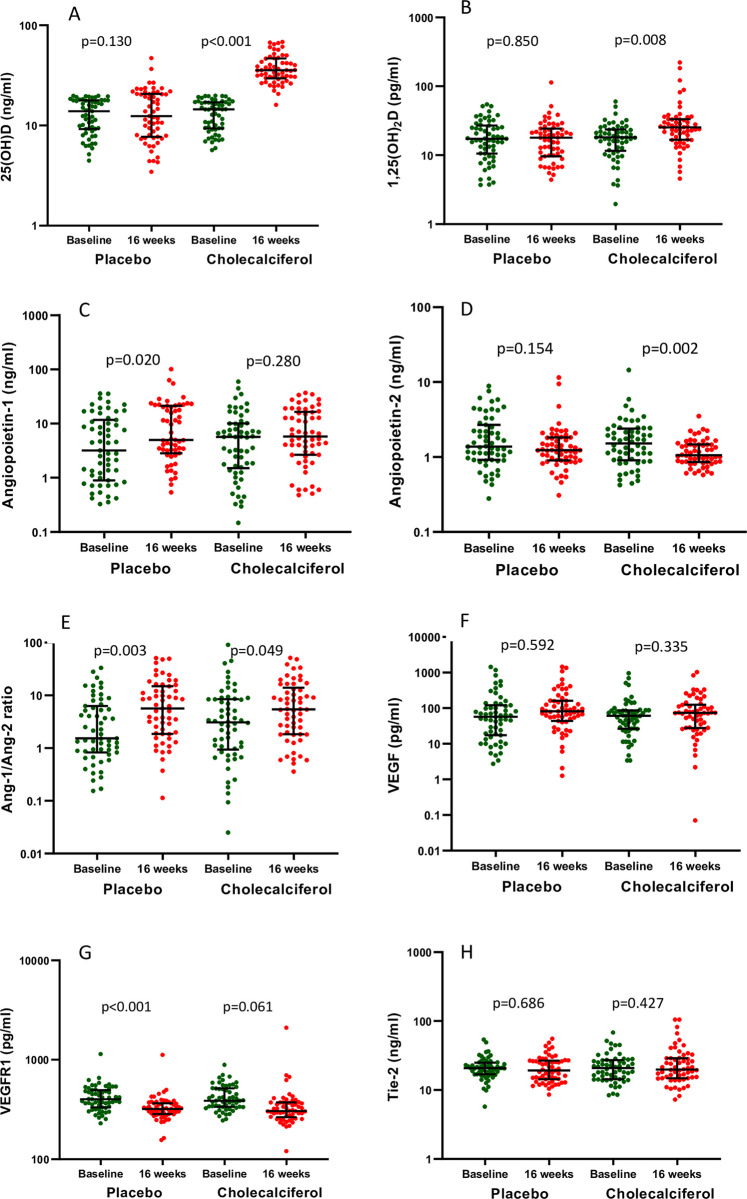
Represents the levels of biomarkers as baseline and follow up in placebo and cholecalciferol group. A. 25 (OH) vitamin D, B. 1,25 (OH)_2_ vitamin D, C. Angiopoietin -1, D. Angiopoietin -2, E. Angiopoietin-1/ Angiopoietin-2 ratio, F. VEGF, G. VEGFR, and H. Tie-2.

Table 3 and Fig 2 shows the within and between group difference of biomarkers as well as the levels of biomarkers at baseline and follow-up in placebo and cholecalciferol group. Distribution of biomarkers at baseline and follow-up has been shown in S1 Fig. At 16 weeks, Ang-2 levels decreased in cholecalciferol group (mean difference: -0.73 ng/ml; 95% CI, -1.25 to -0.20, *p* = 0.002) whereas the levels remained unchanged in the placebo group (mean difference -0.46 ng/ml, 95% CI, -1.09 to 0.17, *p* = 0.154). However, there was no significant between group difference at 16 weeks (-0.27 ng/ml; 95%CI: -1.09 to 0.55*; p* = 0.624; Table 3). Serum Ang-1 levels did not change significantly in cholecalciferol group (mean change: 1.36 ng/ml; 95% CI: -2.39 to 5.11*; p* = 0.280; Table 3) while in placebo group showed an increasing trend (mean change: 5.63 ng/ml; 95%CI: 0.51 to 10.75; *p* = 0.020). Further, the difference in change in the Ang-1 levels between the two groups was not significant (-4.27 ng/ml; 95%CI: -10.56 to 2.01*; p* = 0.445). Serum VEGR levels decreased in placebo group [mean change: -87.16 pg/ml; 95%CI: -131.89 to -42.44; *p*<0.001] but no change in cholecalciferol group was noted (mean change: -69.77 pg/ml; 95%CI: -142.74 to 3.20; *p* = 0.061; Table 3). The levels of serum VEGF and Tie-2 remained unchanged in cholecalciferol as well as placebo groups (Table 3). Further, we analysed the Ang-1/Ang-2 ratio but did not found any significant difference in mean change between groups (-3.32, 95% CI: -9.12 to 2.48, p = 0.563, Table 3).

## Discussion

We have shown through this randomized double-blind placebo-controlled trial that oral high-dose cholecalciferol supplementation can correct native vitamin D deficiency in stable non-diabetic, subjects with G3-4 CKD and induce a favorable change in FMD, a marker of endothelial function [14]. The main intention of the present study was to evaluate the effect of oral cholecalciferol supplementation on serum levels of angiogenic markers in subjects with CKD. Overall, we found no significant effects of a high dose of oral cholecalciferol supplementation on angiogenic markers in these subjects. Although the serum level of Ang-2 was reduced in the cholecalciferol group, the intergroup difference was not significant. There was no effect on the levels of other angiogenic markers such as Ang-1, VEGFR, VEGF, and Tie-2.

Patients with CKD show impaired balance of angiogenic factors caused by an elevation in the circulating level of Ang-2, and reduction in level of Ang-1 [12]. Reduced Ang-1/Ang-2 ratio is a strong predictor of long-term mortality in CKD patients [16]. We found that cholecalciferol supplementation significantly reduced serum levels of Ang-2, as also shown in a recent study that reported a reduction in circulating levels of VEGF-A and Ang-2 in breast cancer patients after treatment with cholecalciferol supplementation [17]. We did not find any difference in the Ang-1/Ang-2 ratio between the two groups.

*In vitro* studies have also confirmed that treatment with vitamin D analogues decreased the expression of Ang-2 in cell lines [18, 19]. In fact, some studies suggested that vitamin D supplementation could inhibit angiogenesis in tumor cells [7, 20, 21]. However, endothelial cells within the tumors are not the same as in normal tissues. In fact, vitamin D shows pro-angiogenic effects in other disorders like preeclampsia, an early stage of CKD, and CVD [8, 14]. Therefore, it is important to understand whether the antiangiogenic effect of vitamin D occurs in both tumor and normal tissues and whether these effects occur by direct or indirect actions on the signalling of angiogenesis. It is well known that Ang-1 is a key regulator of angiogenesis, and stabilizes endothelial cells, but adenoviral delivery of Ang-1 induced inflammatory and fibrotic responses in a mouse folic acid-induced nephrotoxicity model [22]. However, none of the studies has shown the effect of cholecalciferol supplementation on Ang-1 and Tie-2 except this current study and, results suggested that a high dose of oral cholecalciferol has no significance effect on these angiogenic markers.

The interaction between vitamin D and angiogenesis balance has been studied in other conditions. Oral vitamin D_3_ supplementation reduces the risk for preeclampsia, a state of anti-angiogenesis [23–25]. Irani et al. demonstrated a significant decrease in VEGF levels in vitamin D-deficient patients with polycystic ovary syndrome after vitamin D_3_ supplementation [26]. There are some observational studies which reported the high circulating VEGF-A in CKD and diabetic nephropathy and a lower Ang-1/VEGF-A ratio is in patients with CKD [27, 28]. VEGF levels were detected to be increased [29], decreased [30], or did not differ between patients with CKD and healthy controls [31]. But none of these studies has analysed the association or effect of vitamin D on VEGF levels. In our study, VEGF levels remained unchanged in both the groups suggesting that oral cholecalciferol supplementation did not significantly alter the expression of VEGF in patients with early CKD. Interestingly, VEGFR levels significantly declined in the placebo group, but remain unchanged in the cholecalciferol group. The significance of this finding is unclear.

The major strength of our study is the setting of a randomized, double-blind, placebo-controlled trial, homogenous study populations, and adequate sample size to estimate the primary outcome. The relatively short duration, analysis at only two time points, exclusion of subjects with diabetes and the post-hoc nature of the secondary analysis are an important limitation of this study. The study may not be significantly powered to detect differences in outcome parameters for this secondary analysis.

**In conclusion,** our data shows the changes in Ang-1, Ang-2 and Ang-1/Ang-2 ratio after high dose oral cholecalciferol supplementation in patients with non-diabetic G3-4 CKD. The data suggests changes in circulating levels of angiogenic markers which needs to be confirmed through an adequately powered study.

## Supporting information

S1 Checklist CONSORT 2010 checklist of information to include when reporting a randomised trial*. (DOC)

S1 Fig Showing the distribution of biomarkers at baseline and follow-up. (PDF)

S1 File (DOC)
