# Supplementary figures and images for: Cholecalciferol supplementation and angiogenic markers in chronic kidney disease
Source: PLoS One. 2022 Jun 3;17(6):e0268946. doi: 10.1371/journal.pone.0268946 (PMC9165782; doi:10.1371/journal.pone.0268946)

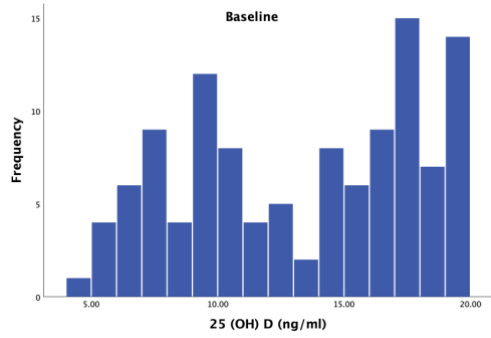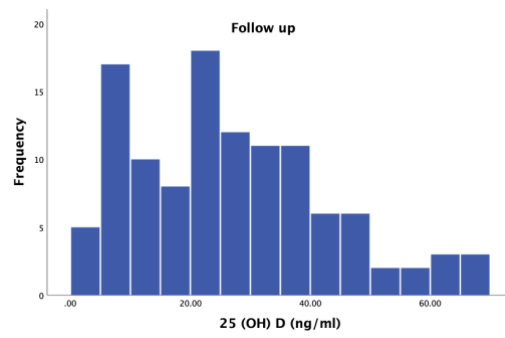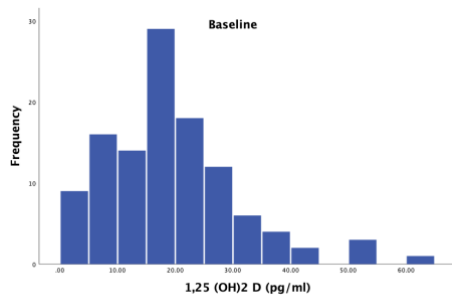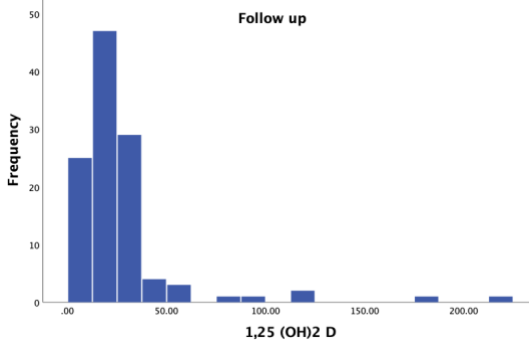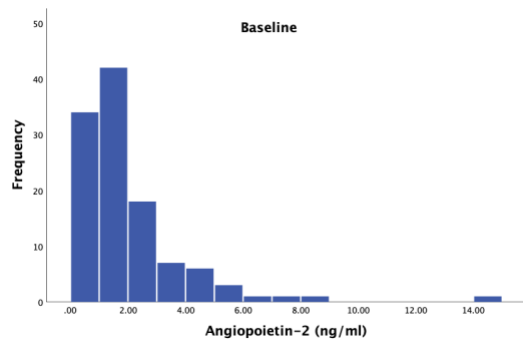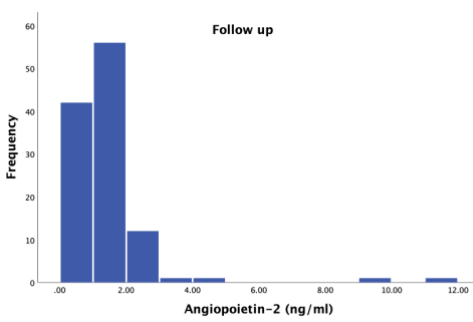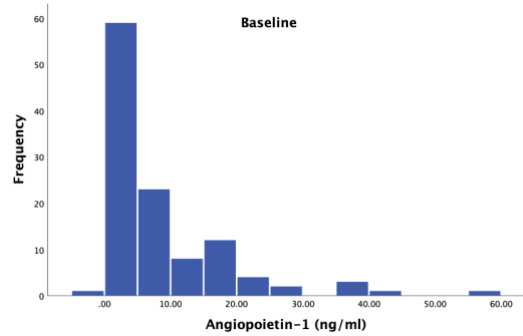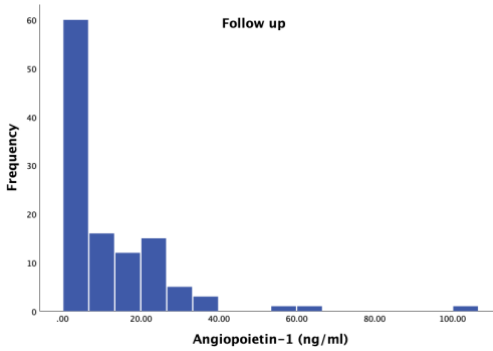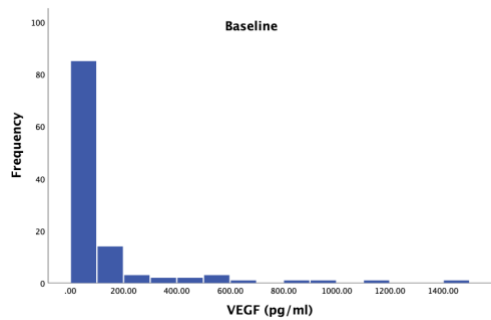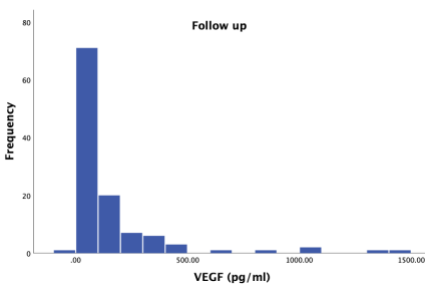

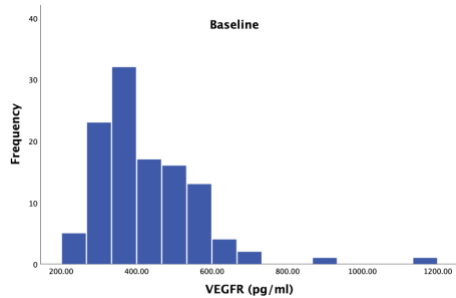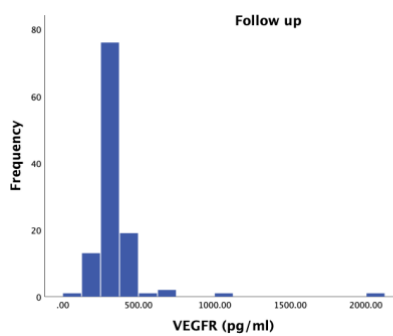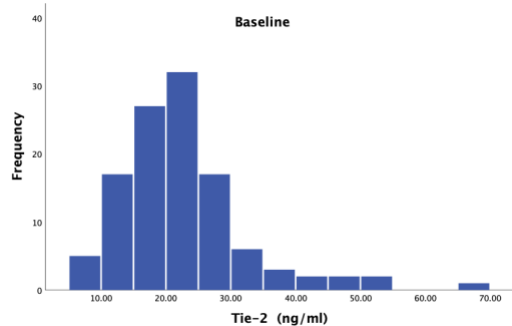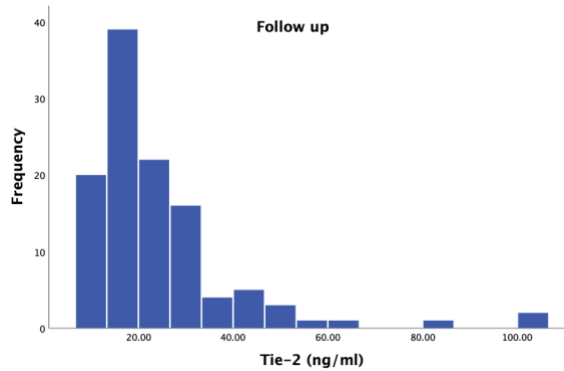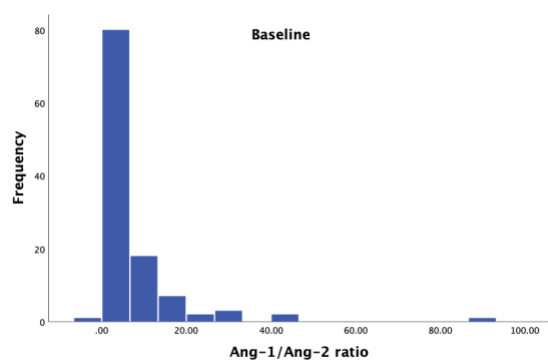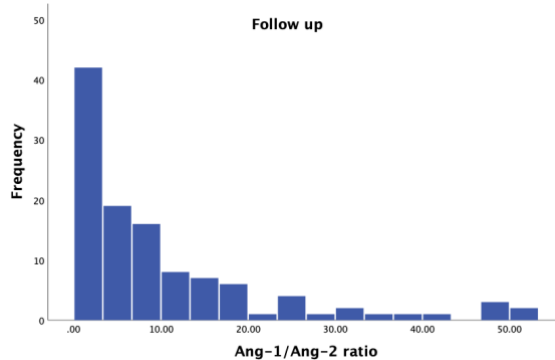

Supplement: S1 Fig — (PDF) [file pone.0268946.s002.pdf]
