# Supplementary material for: Cholecalciferol supplementation and angiogenic markers in chronic kidney disease
Source: PLoS One. 2022 Jun 3;17(6):e0268946. doi: 10.1371/journal.pone.0268946 (PMC9165782; doi:10.1371/journal.pone.0268946)
Supplement: S1 File — (DOC) [file pone.0268946.s003.doc]

**DEPARTMENT OF BIOTECHNOLOGY**

**R & D PROJECT**

**Impact of vitamin D supplementation on vascular function and oxidative stress in patients with chronic kidney disease**

# SUBMITTED BY

## DR. VIVEKANAND JHA

# Professor; Department of Nephrology

# Head; Department of Translation and Regenerative Medicine

# Postgraduate Institute of Medical Education & Research,

# Chandigarh 160 012

**PROFORMA FOR SUBMISSION OF RESEARCH AND**

**DEVELOPMENT PROJECTS, CREATION OF**

**INFRASTRUCTURAL FACILITIES, CENTRES OF EXCELLENCE**

**IN THE IDENTIFIED AREAS AND DEMONSTRATION PROJECTS**

(To be filled by the applicant)

**PART I : GENERAL INFORMATION**

1. Name of the Institute/University/Organisation submitting the Project Proposal:

**Postgraduate Institute of Medical Education & Research, Chandigarh**

2. State: Chandigarh

3. Status of the Institute: Autonomous Institute
(Ministry of Health and Family Welfare, Govt. of India)

4. Name and designation of the University Director, Postgraduate Institute of
Executive Authority of the Institute/ Medical Education & Research,
forwarding the application: Chandigarh

5. Project Title: **Impact of vitamin D supplementation on vascular function and oxidative stress in patients with chronic kidney disease**

6. Category of the Project: R&D

7. Specific Area
(Please see Annexure - II) : Chronic Disease Biology

8. Duration : Three (3) Years

9. Total Cost (Rs.):

10. Is the project Single Institutional or Multiple-Institutional (S/M) ? :
 Single Institutional

11. If the project is multi-institutional, please furnish the following : Not applicable

**12. Scope of application indicating anticipated product and processes**

Vitamin D deficiency has been shown to be associated with increased inflammation, oxidative stress and immune dysfunction. Vitamin D deficiency is nearly universal in CKD patients. It might be assumed that supplementation might mitigate the CV risk of CKD by favorably influencing these parameters. This project has been designed to evaluate the role of oral vitamin D3 supplementation in chronic kidney disease stage 3 and 4 patients. It might be assumed that supplementation might mitigate the CV risk of CKD by favorably influencing these parameters. However no studies thus far have investigated the effect of native vitamin D repletion on CV end points in CKD patients. In our study vitamin D supplementation, if proven to be effective, may significantly reduce CV morbidity and mortality in CKD and address a major public health problem. We also plan to explore the mechanistic basis of this benefit through a comprehensive analysis of oxidative stress, inflammation, endothelial functions.

**13. Project Summary**

CVD is a significant cause of morbidity and mortality in patients with CKD. Vitamin D deficiency is common in CKD11-13, and evidence from observational studies suggests an association between vitamin D deficiency and mortality both in predialysis CKD patients and in patients on dialysis therapy14-16. Moreover, treatment with vitamin D may offer a survival advantage in CKD patients16,17.

We propose to test in a randomized, double-blind, placebo-controlled trial if oral vitamin D3 supplementation over 16 weeks in 108 pre-dialysis CKD stage 3-4 patients with 25 (OH) D deficiency will result in a 40% improvement in endothelial function measured by a non-invasive ultrasound assessment of brachial artery FMD as a response forearm ischaemia.

Vitamin D is inexpensive and easily administered in outpatient clinics. Currently vitamin D measurement is not recommended by international guidelines on CKD management. If proven to be effective, we believe this may significantly reduce CV morbidity and mortality in CKD and address a major public health problem.

**PART III : TECHNICAL DETAILS**

# 16. Introduction

**16.1 Origin of the proposal**

Chronic kidney disease (CKD) is a global public health problem with an estimated prevalence of 8-11% of the adult population in western countries1;2. In absence of registry data the prevalence of CKD in India cannot be accurately determined. Regional studies have estimated it be around 0.79-1.4% of adult population3;4. Patients with CKD are at a considerable risk of death from cardiovascular disease (CVD). CV deaths are 15-30 times higher in patients with CKD than in the age-matched general population5;6. Traditional risk factors for CVD such as hyperlipidemia, hypertension and obesity do not adequately explain this disproportionate increase in risk, and non-traditional risk factors such as inflammation, oxidative stress and altered immune function have been highlighted in recent years7-10. Vitamin D deficiency has been shown to be associated with increased inflammation and oxidative stress. Vitamin D deficiency is nearly universal in CKD patients. It might be assumed that supplementation might mitigate the CV risk of CKD by favorably influencing these parameters. However no studies thus far have investigated the effect of native vitamin D repletion on CV end points in CKD patients.

We aim to demonstrate an improvement in endothelial functions with 16 weeks of oral vitamin D3 therapy in pre-dialysis CKD stage 3-4 (eGFR 15-60 ml/min1.73m2) patients with 25 (OH) D deficiency. We propose that the improvement in endothelial and immune functions is related to reduction in oxidative stress and inflammation, and intend to test these.

**16.2 Rationale**

CKD is a global public health problem, including India. We along with others have shown a graded relationship between CVD and estimated GFR (eGFR) 18;19. Recent evidence points to the important role of inflammation, oxidative stress and vitamin D deficiency as modifiable CV risk factors in CKD8;20;21. Vitamin D deficiency is common in the Indian general population with reported prevalence rates of up to 83%. Female gender, pregnancy, urban lifestyle, poor diet and socio-economic factors have been implicated as a cause of hypovitaminosis D in India22;23. We have shown 77% prevalence of 25 (OH)D deficiency [25 (OH)D <15ng/ml] and a further 22% 25 (OH)D insufficiency [25 (OH)D=15-30ng/ml] in a group of 100 north Indian predialysis CKD patients under follow-up at PGIMER11. Furthermore, patients with kidney disease are unable to synthesize active 1,25 (OH)2 D, make them particularly vulnerable to deleterious effects of hypovitaminosis D.

CVD is a significant cause of morbidity and mortality in patients with CKD. Vitamin D deficiency is common in CKD11-13, and evidence from observational studies suggests an association between vitamin D deficiency and mortality both in predialysis CKD patients and in patients on dialysis therapy14-16. Moreover, treatment with vitamin D may offer a survival advantage in CKD patients16,17.

We propose to test in a randomized, double-blind, placebo-controlled trial if oral vitamin D3 supplementation over 16 weeks in 108 pre-dialysis CKD stage 3-4 patients with 25 (OH) D deficiency will result in a 40% improvement in endothelial function measured by a non-invasive ultrasound assessment of brachial artery FMD as a response forearm ischaemia.

- 1. **Objectives**

We aim to demonstrate an improvement in endothelial functions with 16 weeks of oral vitamin D3 therapy in pre-dialysis CKD stage 3-4 (eGFR 15-60 ml/min1.73m^2^) patients with 25 (OH) D deficiency. We propose that the improvement in endothelial and immune functions is related to reduction in oxidative stress and inflammation, and intend to test these hypotheses through the following specific aims:

**Specific Aim # 1:**

To investigate if directly observed 300,000 IU oral Colecalciferol (vitamin D3) at 0 and 8 weeks results in a 40% improvement in endothelial function in CKD stage 3 and 4 patients with vitamin D deficiency [serum 25(OH) D levels< 50 nmol/L] over 16 weeks.

**Specific Aim # 2:**

To investigate if oral supplementation of Colecalciferol in vitamin D deficient CKD stage 3 and 4 patients, improves systemic inflammation and oxidative stress over 16 weeks.

These specific aims will be tested in a randomized, double-blind, placebo-controlled trial involving 120 patients assigned to two equal groups to receive placebo or Colecalciferol therapy. Brachial artery Flow Mediated Dilatation (FMD) will be used to assess the endothelial function at baseline and at 16 week follow-up. In addition the following biomarkers will be measured pre and post vitamin D therapy:

1. Serum High sensitivity C-reactive protein (hsCRP) and Interleukin-6 for inflammation,

2. Plasma F-2 Isoprostanes for lipid peroxidation;

3. Serum Fibroblast growth factor-23 (FGF-23) levels

4. Plasma Von Willebrand factor and Serum E-selectin for endothelial cell activation and injury, respectively.

If proven to be effective, native vitamin D supplementation can prove to be an inexpensive therapeutic strategy for a significant public health problem of cardiovascular disease in CKD.

**16.5. Review of current status of research and development in the subject**

Large epidemiological studies have shown a direct independent relationship between vitamin D deficiency and CVD risk and mortality in the general population^15;24;25^. In patients with CKD, vitamin D deficiency is associated with increased CV mortality. Moreover, retrospective studies in End stage kidney disease (ESRD) have shown an improved CV mortality in patients treated with vitamin D (adjusted OR=0.60, 95% CI 0.37-0.91)^16^. Impaired endothelial function and central arterial stiffness are a hallmark feature of patients with CKD^26;27;28^, possibly as a consequence of increased oxidative stress^26^. Vitamin D improves acetylcholine induced relaxation of blood vessels in animal experiments, both in hypertensive and renal failure rats models^29;30^. This effect has been proposed to be due to a direct action of vitamin D on endothelial nitric oxide release^30^. Indeed in haemodialysis patients, vitamin D levels negatively correlate with brachial artery FMD and central arterial stiffness^31^, the latter possibly driven by an effect of FGF23 on vascular calcification. FMD was also inversely related to systemic inflammation in our patient population, thus suggesting that nutritional vitamin D deficiency is related to vascular disease in CKD. Most importantly, vitamin D supplementation has been shown to improve endothelial function in patients with type II diabetes mellitus after only 8 weeks of therapy^32^. Thus vitamin D directly affects endothelial cell responsiveness. *However, no studies so far have shown an improvement in endothelial function and central arterial stiffness in pre-dialysis CKD patients treated with native vitamin D in a randomised placebo-controlled trial.*

***Vitamin D, inflammation and oxidative stress***

Several reports have ascribed an immunoregulatory function to vitamin D. Epidemiological data shows that polymorphism of the Vitamin D receptor or CYP27B1 (1α-hydroxylase)] increases susceptibility to autoimmune diseases^33^, which is in keeping with epidemiological studies in humans demonstrating associations between Vitamin D insufficiency and autoimmune diseases, including type 1 diabetes^34;35^. Mechanistically, Vitamin D therapy modulates T cell function, promoting, in collaboration with interleukin (IL)-2, development of regulatory T cells (Tregs)^36^, inhibiting dendritic cell (DC) maturation and attenuating antigen presenting capacity^34^. Indeed, general population surveys show an inverse relationship between vitamin D levels and systemic inflammation measured by C-reactive protein^37^.

Oxidative stress results in impaired endothelial function in CKD^26^. Vitamin D inhibits oxidative stress ex-vivo. In human umbilical vein endothelial cell culture studies, vitamin D has been shown to inhibit advanced glycation endproduct (AGE) induced IL6 levels and NF-kβ activity as well as promoting endothelial nitric oxide synthesis^38^. Thus, vitamin D may play an important role in inhibiting the harmful effects of oxidative stress on nitric oxide release by endothelial cells. *However, the effect of vitamin D repletion on oxidative stress and inflammation has not been tested in human trials.*

***Vitamin D and FGF-23***

Vascular disease and bone mineral disorders are intricately linked in CKD. High serum phosphate level has been recognised as a major player promoting in vascular calcification and thereby mortality in patients with CKD^39-^42. Indeed, drugs to inhibit phosphate absorbtion are an important therapeutic tool in patients with CKD. Vitamin D, serum parathyroid hormone (PTH) and FGF-23 are key endocrine components regulating serum phosphate levels and possibly subsequent vascular calcification. FGF-23 is released from osteoblasts in response to high serum phosphate^43^. FGF-23 promote phosphate excretion and inhibits active 1,25 (OH)_2_ D synthesis^44^. Serum FGF-23 is directly associated with left ventricular hypertrophy and vascular calcification in CKD, where it is thought to play a regulatory role^45;46^. FGF-23 is also an independent predictor of mortality in ESRD patients^47^. *Thus FGF-23 is an important modulator of vascular function in CKD but the role of native vitamin D supplementation on FGF23 expression and subsequent effect on vascular function has yet to be determined.*

**References:**

1. Coresh J, Astor BC, Greene T, Eknoyan G, Levey AS: Prevalence of chronic kidney disease and decreased kidney function in the adult US population: Third National Health and Nutrition Examination Survey. *Am J Kidney Dis* 41:1-12, 2003

2. Stevens PE, O'Donoghue DJ, de LS, Van VJ, Klebe B, Middleton R, Hague N, New J, Farmer CK: Chronic kidney disease management in the United Kingdom: NEOERICA project results. *Kidney Int* 72:92-99, 2007

3. Agarwal SK, Dash SC, Irshad M, Raju S, Singh R, Pandey RM: Prevalence of chronic renal failure in adults in Delhi, India. *Nephrol Dial Transplant* 20:1638-1642, 2005

4. Mani MK: Experience with a program for prevention of chronic renal failure in India. *Kidney Int Suppl*S75-S78, 2005

5. Foley RN, Parfrey PS, Sarnak MJ: Clinical epidemiology of cardiovascular disease in chronic renal disease. *Am J Kidney Dis* 32:S112-S119, 1998

6. Parfrey PS, Foley RN: The clinical epidemiology of cardiac disease in chronic renal failure. *J Am Soc Nephrol* 10:1606-1615, 1999

7. Arici M, Walls J: End-stage renal disease, atherosclerosis, and cardiovascular mortality: is C-reactive protein the missing link? *Kidney Int* 59:407-414, 2001

8. Cachofeiro V, Goicochea M, de Vinuesa SG, Oubina P, Lahera V, Luno J: Oxidative stress and inflammation, a link between chronic kidney disease and cardiovascular disease. *Kidney Int Suppl*S4-S9, 2008

9. Himmelfarb J, Stenvinkel P, Ikizler TA, Hakim RM: The elephant in uremia: oxidant stress as a unifying concept of cardiovascular disease in uremia. *Kidney Int* 62:1524-1538, 2002

10. Menon V, Greene T, Wang X, Pereira AA, Marcovina SM, Beck GJ, Kusek JW, Collins AJ, Levey AS, Sarnak MJ: C-reactive protein and albumin as predictors of all-cause and cardiovascular mortality in chronic kidney disease. *Kidney Int* 68:766-772, 2005

11. Jabbar Z, Aggarwal PK, Chandel N, Kohli HS, Gupta KL, Sakhuja V, Jha V: High prevalence of vitamin D deficiency in north Indian adults is exacerbated in those with chronic kidney disease. *Nephrology (Carlton )* 14:345-349, 2009

12. Manghat P, Fraser WD, Wierzbicki AS, Fogelman I, Goldsmith DJ, Hampson G: Fibroblast growth factor-23 is associated with C-reactive protein, serum phosphate and bone mineral density in chronic kidney disease. *Osteoporos Int* 2009

13. Mehrotra R, Kermah D, Budoff M, Salusky IB, Mao SS, Gao YL, Takasu J, Adler S, Norris K: Hypovitaminosis D in chronic kidney disease. *Clin J Am Soc Nephrol* 3:1144-1151, 2008

14. Inaguma D, Nagaya H, Hara K, Tatematsu M, Shinjo H, Suzuki S, Mishima T, Kurata K: Relationship between serum 1,25-dihydroxyvitamin D and mortality in patients with pre-dialysis chronic kidney disease. *Clin Exp Nephrol* 12:126-131, 2008

15. Mehrotra R, Kermah DA, Salusky IB, Wolf MS, Thadhani RI, Chiu YW, Martins D, Adler SG, Norris KC: Chronic kidney disease, hypovitaminosis D, and mortality in the United States. *Kidney Int* 76:977-983, 2009

16. Wolf M, Shah A, Gutierrez O, Ankers E, Monroy M, Tamez H, Steele D, Chang Y, Camargo CA, Jr., Tonelli M, Thadhani R: Vitamin D levels and early mortality among incident hemodialysis patients. *Kidney Int* 72:1004-1013, 2007

17. Kovesdy CP, Ahmadzadeh S, Anderson JE, Kalantar-Zadeh K: Association of activated vitamin D treatment and mortality in chronic kidney disease. *Arch Intern Med* 168:397-403, 2008

18. Banerjee D, Contreras G, Jaraba I, Carvalho D, Ortega L, Carvalho C, Pezon C, Rosenthal SP, De La RN, Vemuri N, Cherla G, Nahar N: Chronic kidney disease stages 3-5 and cardiovascular disease in the veterans affairs population. *Int Urol Nephrol* 41:443-451, 2009

19. Go AS, Chertow GM, Fan D, McCulloch CE, Hsu CY: Chronic kidney disease and the risks of death, cardiovascular events, and hospitalization. *N Engl J Med* 351:1296-1305, 2004

20. Wu-Wong JR: Endothelial dysfunction and chronic kidney disease: treatment options. *Curr Opin Investig Drugs* 9:970-982, 2008

21. Wu-Wong JR: Potential for vitamin D receptor agonists in the treatment of cardiovascular disease. *Br J Pharmacol* 158:395-412, 2009

22. Sahu M, Bhatia V, Aggarwal A, Rawat V, Saxena P, Pandey A, Das V: Vitamin D deficiency in rural girls and pregnant women despite abundant sunshine in northern India. *Clin Endocrinol (Oxf)* 70:680-684, 2009

23. Zargar AH, Ahmad S, Masoodi SR, Wani AI, Bashir MI, Laway BA, Shah ZA: Vitamin D status in apparently healthy adults in Kashmir Valley of Indian subcontinent. *Postgrad Med J* 83:713-716, 2007

24. Dobnig H, Pilz S, Scharnagl H, et al. Independent association of low serum 25-hydroxyvitamin D and 1,25 dihydroxyvitamin D levels with all-cause and cardiovascular mortality. Arch Intern Med 2008; 168:1340–1349.

25. Martins D, Wolf M, Pan D, Zadshir A, Tareen N, Thadhani R, Felsenfeld A, Levine B, Mehrotra R, Norris K: Prevalence of cardiovascular risk factors and the serum levels of 25-hydroxyvitamin D in the United States: data from the Third National Health and Nutrition Examination Survey. *Arch Intern Med* 167:1159-1165, 2007

26. Endemann DH, Schiffrin EL: Endothelial dysfunction. *J Am Soc Nephrol* 15:1983-1992, 2004

27. London GM, Marchais SJ, Guerin AP, Metivier F, Adda H: Arterial structure and function in end-stage renal disease. *Nephrol Dial Transplant* 17:1713-1724, 2002

28. Thambyrajah J, Landray MJ, McGlynn FJ, Jones HJ, Wheeler DC, Townend JN: Abnormalities of endothelial function in patients with predialysis renal failure. *Heart* 83:205-209, 2000

29. Borges AC, Feres T, Vianna LM, Paiva TB: Effect of cholecalciferol treatment on the relaxant responses of spontaneously hypertensive rat arteries to acetylcholine. *Hypertension* 34:897-901, 1999

30. Wu-Wong JR, Noonan W, Nakane M, Brooks KA, Segreti JA, Polakowski JS, Cox B: Vitamin d receptor activation mitigates the impact of uremia on endothelial function in the 5/6 nephrectomized rats. *Int J Endocrinol* 2010:625852, 2010

31. London GM, Guerin AP, Verbeke FH, Pannier B, Boutouyrie P, Marchais SJ, Metivier F: Mineral metabolism and arterial functions in end-stage renal disease: potential role of 25-hydroxyvitamin D deficiency. *J Am Soc Nephrol* 18:613-620, 2007

32. Sugden JA, Davies JI, Witham MD, Morris AD, Struthers AD: Vitamin D improves endothelial function in patients with Type 2 diabetes mellitus and low vitamin D levels. *Diabet Med* 25:320-325, 2008

33. Valdivielso JM, Fernandez E: Vitamin D receptor polymorphisms and diseases. *Clin Chim Acta* 371:1-12, 2006

34. Bouillon R, Carmeliet G, Verlinden L, van EE, Verstuyf A, Luderer HF, Lieben L, Mathieu C, Demay M: Vitamin D and human health: lessons from vitamin D receptor null mice. *Endocr Rev* 29:726-776, 2008

35. Hypponen E, Laara E, Reunanen A, Jarvelin MR, Virtanen SM: Intake of vitamin D and risk of type 1 diabetes: a birth-cohort study. *Lancet* 358:1500-1503, 2001

36. Jeffery LE, Burke F, Mura M, Zheng Y, Qureshi OS, Hewison M, Walker LS, Lammas DA, Raza K, Sansom DM: 1,25-Dihydroxyvitamin D3 and IL-2 combine to inhibit T cell production of inflammatory cytokines and promote development of regulatory T cells expressing CTLA-4 and FoxP3. *J Immunol* 183:5458-5467, 2009

37. Timms PM, Mannan N, Hitman GA, Noonan K, Mills PG, Syndercombe-Court, Aganna E, Price CP, Boucher BJ: Circulating MMP9, vitamin D and variation in the TIMP-1 response with VDR genotype: mechanisms for inflammatory damage in chronic disorders? *QJM* 95:787-796, 2002

38. Talmor Y, Golan E, Benchetrit S, Bernheim J, Klein O, Green J, Rashid G: Calcitriol blunts the deleterious impact of advanced glycation end products on endothelial cells. *Am J Physiol Renal Physiol* 294:F1059-F1064, 2008

39. Block GA, Hulbert-Shearon TE, Levin NW, Port FK: Association of serum phosphorus and calcium x phosphate product with mortality risk in chronic hemodialysis patients: a national study. *Am J Kidney Dis* 31:607-617, 1998

40. Giachelli CM, Jono S, Shioi A, Nishizawa Y, Mori K, Morii H: Vascular calcification and inorganic phosphate. *Am J Kidney Dis* 38:S34-S37, 2001

41. Goodman WG, Goldin J, Kuizon BD, Yoon C, Gales B, Sider D, Wang Y, Chung J, Emerick A, Greaser L, Elashoff RM, Salusky IB: Coronary-artery calcification in young adults with end-stage renal disease who are undergoing dialysis. *N Engl J Med* 342:1478-1483, 2000

42. London GM, Pannier B, Marchais SJ, Guerin AP: Calcification of the aortic valve in the dialyzed patient. *J Am Soc Nephrol* 11:778-783, 2000

43. Antoniucci DM, Yamashita T, Portale AA: Dietary phosphorus regulates serum fibroblast growth factor-23 concentrations in healthy men. *J Clin Endocrinol Metab* 91:3144-3149, 2006

44. Shimada T, Hasegawa H, Yamazaki Y, Muto T, Hino R, Takeuchi Y, Fujita T, Nakahara K, Fukumoto S, Yamashita T: FGF-23 is a potent regulator of vitamin D metabolism and phosphate homeostasis. *J Bone Miner Res* 19:429-435, 2004

45. Gutierrez OM, Januzzi JL, Isakova T, Laliberte K, Smith K, Collerone G, Sarwar A, Hoffmann U, Coglianese E, Christenson R, Wang TJ, deFilippi C, Wolf M: Fibroblast growth factor 23 and left ventricular hypertrophy in chronic kidney disease. *Circulation* 119:2545-2552, 2009

46. Nasrallah MM, El-Shehaby AR, Salem MM, Osman NA, El SE, Sharaf El Din UA: Fibroblast growth factor-23 (FGF-23) is independently correlated to aortic calcification in haemodialysis patients. *Nephrol Dial Transplant* 2010

47. Gutierrez OM, Mannstadt M, Isakova T, Rauh-Hain JA, Tamez H, Shah A, Smith K, Lee H, Thadhani R, Juppner H, Wolf M: Fibroblast growth factor 23 and mortality among patients undergoing hemodialysis. *N Engl J Med* 359:584-592, 2008

48. Chandra P, Binongo JN, Ziegler TR, Schlanger LE, Wang W, Someren JT, Tangpricha V: Cholecalciferol (vitamin D3) therapy and vitamin D insufficiency in patients with chronic kidney disease: a randomized controlled pilot study. *Endocr Pract* 14:10-17, 2008

49. Deanfield J, Donald A, Ferri C, Giannattasio C, Halcox J, Halligan S, Lerman A, Mancia G, Oliver JJ, Pessina AC, Rizzoni D, Rossi GP, Salvetti A, Schiffrin EL, Taddei S, Webb DJ: Endothelial function and dysfunction. Part I: Methodological issues for assessment in the different vascular beds: a statement by the Working Group on Endothelin and Endothelial Factors of the European Society of Hypertension. *J Hypertens* 23:7-17, 2005

50. Sidhu JS, Newey VR, Nassiri DK, Kaski JC: A rapid and reproducible on line automated technique to determine endothelial function. *Heart* 88:289-292, 2002

51. Kadiiska MB, Gladen BC, Baird DD, Germolec D, Graham LB, Parker CE, Nyska A, Wachsman JT, Ames BN, Basu S, Brot N, Fitzgerald GA, Floyd RA, George M, Heinecke JW, Hatch GE, Hensley K, Lawson JA, Marnett LJ, Morrow JD, Murray DM, Plastaras J, Roberts LJ, Rokach J, Shigenaga MK, Sohal RS, Sun J, Tice RR, Van Thiel DH, Wellner D, Walter PB, Tomer KB, Mason RP, Barrett JC: Biomarkers of oxidative stress study II: are oxidation products of lipids, proteins, and DNA markers of CCl4 poisoning? *Free Radic Biol Med* 38:698-710, 2005

52. Corretti MC, Anderson TJ, Benjamin EJ, Celermajer D, Charbonneau F, Creager MA, Deanfield J, Drexler H, Gerhard-Herman M, Herrington D, Vallance P, Vita J, Vogel R: Guidelines for the ultrasound assessment of endothelial-dependent flow-mediated vasodilation of the brachial artery: a report of the International Brachial Artery Reactivity Task Force. *J Am Coll Cardiol* 39:257-265, 2002

**16.6. Relevance of the proposed project and expected outcome**

Vitamin D deficiency has been shown to be associated with increased inflammation, oxidative stress and immune dysfunction. Vitamin D deficiency is nearly universal in CKD patients. It might be assumed that supplementation might mitigate the CV risk of CKD by favorably influencing these parameters. This project has been designed to evaluate the role of oral vitamin D3 supplementation in chronic kidney disease stage 3 and 4 patients. It might be assumed that supplementation might mitigate the CV risk of CKD by favorably influencing these parameters. However no studies thus far have investigated the effect of native vitamin D repletion on CV end points in CKD patients. In our study vitamin D supplementation, if proven to be effective, we believe this may significantly reduce CV morbidity and mortality in CKD and address a major public health problem

16.7 Preliminary work done by investigator on this problem:

In our previous study we have shown that majority of the CKD patients in our population have insufficient level of vitamin D. We have shown 77% prevalence of 25 (OH)D deficiency [25 (OH)D <15ng/ml] and a further 22% 25 (OH)D insufficiency [25 (OH)D=15-30ng/ml] in a group of 100 north Indian predialysis CKD patients under follow-up at PGIMER (Jabbar et al 2008). In a previous ICMR-funded study, we have also shown a high prevalence of activation of inflammatory markers including the iron regulatory protein, hepcidin in stage IV-V CKD subjects (Jairam et al 2010). We have shown immune dysfunction in the form of alteration in specific T-cell subset, CX3XL1 and CX3CR1 and their correlation with atherosclerosis (Yadav and Jha 2011, Yadav et al 2011).

**18. Workplan**

**18.1 Methodology:**

***Study Population***

Vitamin D naive patients will be recruited from the general nephrology outpatient clinic at PGIMER, Chandigarh. Patients with 25 (OH) D deficiency [(25 (OH) D <20 ng/ml] will be enrolled into the randomisation phase. Standard patient demographics and pertinent clinical history would be obtained upon entry by the investigator. Blood samples will be collected for PTH, hsCRP, IL-6 and FGF-23 measurement. Samples will be stored in -70^0^ C and batch analysed for oxidative stress (F2- isoprostanes) and markers of endothelial injury and activation (Von willebrand factor and E-selectin, respectively). Brachial Artery FMD studies will be performed to assess endothelial function. Central arterial stiffness will be measured by central arterial Pulse Wave Velocity (PWV) and Augmentation Index (AI) using SphygmoCor Pulse Wave Velocity system. At 16 weeks follow-up FMD, PWV, AI and biochemistry, biomarkers of inflammation, oxidative stress, and endothelial function will be repeated.

***Inclusion and exclusion criteria***

Adult (18-70 years) patients with estimated GFR between 15-60 ml/min/1.73 m^2^ (CKD stage 3 and 4) will be recruited. The following will be excluded from the trial:

- Patients with Diabetes mellitus
- Active smokers
- Patients with Hb<8 gm%
- Uncontrolled hypertension
- Patients already on Vitamin D supplementation
- Patients with known malignancies
- Patients with autoimmune conditions
- Patients with heart failure
- Rapidly deteriorating renal function
- Recent Acute coronary syndrome (within the last 3 months)
- Pregnant females

***Patient randomization, blinding, treatment and follow-up***

The dialysis unit Tutor-Technician will oversee the randomization into two equal groups for comparison, blinding and dispensing the medications.

Randomization will be against a computer generated list held in the pharmacy department by the Clinical Trials team and the allocation of medications- active or placebo to individual study subjects will be performed by the Clinical Trials Unit in pharmacy on assignment to the next available entry.

The stages of the trial are summarised below:

i. 120 patients with 25 (OH) D levels < 20 ng/ml will be enrolled, randomized, will enter 2 weeks run in period and then receive 2 directly observed oral doses of 300,000 IU colecalciferol at 0 and 8 weeks or matching placebo (60 patients in each arm). Over 16 weeks, 25 (OH)D >30 ng/ml will be achieved in the active treatment group to ensure vitamin D repletion. This has been achieved in previous reported studies using a similar cumulative dose48.

ii. At 16 weeks blood samples will be collected for repeat biochemistry, markers of oxidative stress, inflammation, FGF-23 and endothelial cell activation and injury as mentioned above. Change in endothelial function and arterial stiffness will be re-measured using brachial artery flow mediated dilatation studies and PWV and AI, respectively.

Changes in the pre and post treatment measurements will be compared in the active and placebo group. The patients will continue to be followed up in the general nephrology clinic during the trial and subsequently upon trial completion.

***Recruitment and retention of participants***

Planning and preparation of the trial would be conducted over the first 4 weeks. About 250 CKD patients/week are seen at PGIMER. 30% of the patients are diabetic and 50% of the patients have a GFR < 45 mL/min/1.73m2. With 90% vitamin D deficiency in our local population and a recruitment rate of 5%, 10 patients can be recruited every week. Therefore total recruitment will be over by 25 weeks. After 16 weeks of cholecalciferol therapy repeat analysis will be performed between weeks 23 to 53. With the short duration of the study and continued follow up of patients in the outpatient clinics, we anticipate > 90% retention rate of the trial participants. The data will be analysed and the findings reported between 53-60 weeks. We are confident that we have sufficient patient’s numbers to recruit and complete the study within the stipulated time period.

***Brachial artery flow mediated dilatation***

Brachial artery flow mediated dilatation (FMD) is a non-invasive, easily reproducible, well validated method of assessing endothelial function by measuring nitric oxide induced vessel dilatation in response to forearm ischaemia49 with online technique of analysis of changes in brachial artery diameter using a vessel image analysis software that virtually eliminates the need for operator intervention for analysis purposes. This method results in significant reduction in mean day to day variability of FMD to 0.5%- 1%50.

The analysis will be performed after an overnight fast for 12 hours. Patients will be asked to refrain from smoking on the day and withhold their medications until after the study. Brachial artery will be scanned with a 5-12 MHz linear transducer, 2-10 cms from the elbow. The ultrasound system is connected to a personal computer equipped with a frame grabber and a vessel image analysis software, which automatically detects and tracks the anterior and posterior wall of the artery within a user defined region of interest, providing real time data acquisition. B mode image acquisition is at 25 frames/sec and vessel diameter changes over cardiac cycle are displayed in real time. A baseline image of the brachial artery will be obtained for 2 minutes, following which a forearm tourniquet is inflated to 300 mmHg for 4.5 minutes to induce distal forearm ischaemia. Brachial artery diameter following reactive hyperemia will be recorded for 5 minutes after tourniquet release. FMD, defined as a percentage increase in brachial artery diameter after tourniquet release, will be automatically calculated by the vessel image analysis software. Sublingual Glycerine trinitrate (400 mcg) will then be administered to the participants and further imaging performed for 5 minutes to determine endothelium independent FMD. The total duration of the test is about 20 minutes with no anticipated side-effects or discomfort to the patient.

***Central arterial stiffness studies using pulse wave velocity and augmentation index***

Arterial stiffness will be assessed non-invasively using a pressure tonometer coupled to a SphygmoCor Pulse Wave Velocity system at baseline. Patients will be asked to avoid food, tobacco and caffeine for at least 2 hours before these visits and will be studied lying supine on an examination couch at the same sitting as the FMD analyses to minimise patient inconvenience. The hemodynamic monitoring would be performed after 15 minutes of rest, in a controlled environment at 22°C. A simultaneously recorded ECG signal (requiring 3 chest leads) will provide an R-wave timing reference. The software will process each set of pressure pulses and ECG waveform data to calculate the mean time difference between the arrivals of the pulse at the two peripheral recording sites on a beat to beat basis. The pulse wave velocity is then calculated using the mean time difference and the arterial path length between the two recording sites. At the same time blood pressure would be recorded over the right arm and a mean of three readings would be considered. Using the same equipment, the radial pulse waveform will be analysed and the corresponding central aortic waveform generated using the SphygmoCor systems software.

***Vitamin D measurement***

25 (OH) D and Serum 1,25 (OH)2 D will be analyzed by Sandwich enzyme-linked immunosorbent assay (ELISA) (IDS Ltd, Fountain Hills, AZ, USA).

***Marker of oxidative stress (plasma f-2 isoprostanes)***

F 2 isoprostanes, also known as 8- iso-prostaglandin F2α, are a series of prostaglandin F2α-like compounds produced by the free radical–catalyzed peroxidation of arachidonic acid independent of the cyclooxygenase. They are present in all body tisuues and biological fluids and provide an accurate quantitative assessment of lipid peroxidation and therefore oxidative stress in vivo. A recent multicentre study, sponsored by National Institute of Health has concluded that F2 isoprostanes is the most accurate assessment of oxidative stress and should be adopted as gold standard for measurement of oxidative stress in-vivo^51^.

We will use 8 isoprostane EIA kit (Caymen Chemicals, Ann anbor, MI) for quantification of F2 isoprostanes in patient plasma. Plasma obtained from the patient will undergo alkaline hydrolysis by adding equal volume of 40% wt/vol KOH and incubating at 40 degrees C for 60 minutes to estimate both free and esterified 8 isoprostanes in serum. Sample will then undergo solid phase extraction (SPE) purification prior to the assay. The intra and interassay coefficient of variation of this assay is 6.4% and 24.3% respectively for the normal range of 8 isoprostanes. The lowest detectable limit of the assay is 2.7 pg/ml.

***Markers of endothelial cell injury and activation***

*Von Willebrand Factor (VWF):* VWF is a glycoprotein that binds to collagen and platelets as a rapid response to endothelial injury. It is secreted almost exclusively by vascular endothelium and is bound to the basement membrane and exists freely in plasma.

We will analyse plasma VWF activity (Instrumentation laboratory, UK). The kit is a latex particle enhanced immunoturbidometric assay to quantify VWF activity in plasma determined by measuring increase in turbidity produced by the agglutination of anti VWF monoclonal antibody bound latex reagent upon admixture with plasma. The intra and inter assay coefficient of variation of this assay is 2.7 and 4.9% respectively.

*E-Selectin:* E-selectin (CD62E), leucocyte adhesion molecule is secreted exclusively by vascular endothelium is response to endothelial cell activation.

Serum E-selectin will be estimated from frozen serum using a human soluble E-selectin (sE-Selectin) Quantikine immunoassay (R&D systems, Minneapolis, USA). The intra and inter assay coefficient of variation for this assay is 5.2% and 7.7% respectively.

***Serum FGF-23 assay***

Serum levels of intact FGF-23 molecule will be determined in samples stored at -70 ^0^ C using a two-site (NH2-terminal/C-terminal) enzyme-linked immunosorbent assay (Immutopics, CA, USA) in duplicates.

***Statistical analysis***

The sample analysis will include age, sex, presence of hypertension, previous CVD, BMI, serum lipids, serum albumin, smoking history, eGFR and medication history such as use of statins. Change in FMD measurements (expressed as percentage) would be compared pre and post treatment between the two groups using independent sample T test or Wilcoxon rank sum test. Changes in parameters will also be compared between the two groups, before and after treatment. Categorical variables will be compared using Chi square test. Unifactorial analysis will test for association between clinical, biochemical and arterial parameters with endothelial function. Pre specified subgroup analysis (based on gender, age, serum 25 (OH)D tertiles, use of statins or renin angiotensin system blockers) will be done using linear model of FMD change.

***Power calculation***

Calculations for this project are based on the brachial artery FMD study. The observed within subject variability of this technique is 0.5-1%. We have followed the guidance on the research application of FMD studies proposed by International Brachial Artery Reactivity Task Force, whilst doing the power calculations52.

We will recruit 120 patients (60 in each arm); assuming a retention rate of 90% and baseline FMD of 4%, this sample size will be required to detect difference of 1.6% (standard deviation 3%) between groups (40% relative improvement) with 80% power and two sided alpha of 0.05.
